# A Novel Bifunctional Fusion Protein (Anti-IL-17A-sST2) Protects against Acute Liver Failure, Modulating the TLR4/MyD88 Pathway and NLRP3 Inflammasome Activation

**DOI:** 10.3390/biomedicines12051118

**Published:** 2024-05-17

**Authors:** Yu Bai, Rongrui Zhou, Xinlei Xie, An Zhu, Yanyang Nan, Tao Wu, Xiaozhi Hu, Zhonglian Cao, Dianwen Ju, Jiajun Fan

**Affiliations:** 1 Department of Biological Medicines and Shanghai Engineering Research Center of Immunotherapeutics, School of Pharmacy, Fudan University, Shanghai 201203, China; 2 Fudan Zhangjiang Institute, Shanghai 201203, China; 3 Shanghai Hailu Biological Technology Co., Ltd., Shanghai 201200, China

**Keywords:** acute liver failure, IL-33/ST2, IL-17A, LPS/D-GalN, TLR4, NLRP3

## Abstract

Acute liver failure (ALF) is a serious inflammatory disorder with high mortality rates, which poses a significant threat to human health. The IL-33/ST2 signal is a crucial regulator in inflammation responses associated with lipopolysaccharide (LPS)-induced macrophages. The IL-17A signaling pathway promotes the release of chemokines and inflammatory cytokines, recruiting neutrophils and T cells under LPS stimulation, thus facilitating inflammatory responses. Here, the potential therapeutic benefits of neutralizing the IL-17A signal and modulating the IL-33/ST2 signal in ALF were investigated. A novel dual-functional fusion protein, anti-IL-17A-sST2, was constructed, which displayed high purity and biological activities. The administration of anti-IL-17A-sST2 resulted in significant anti-inflammatory benefits in ALF mice, amelioration of hepatocyte necrosis and interstitial congestion, and reduction in TNF-α and IL-6. Furthermore, anti-IL-17A-sST2 injection downregulated the expression of TLR4 and NLRP3 as well as important molecules such as MyD88, caspase-1, and IL-1β. The results suggest that anti-IL-17A-sST2 reduced the secretion of inflammatory factors, attenuated the inflammatory response, and protected hepatic function by regulating the TLR4/MyD88 pathway and inhibiting the NLRP3 inflammasome, providing a new therapeutic approach for ALF.

## 1. Introduction

Acute liver failure (ALF) is a rapidly progressing inflammatory disease that typically occurs due to sudden hepatic dysfunction, characterized by massive hepatocyte death and excessive inflammatory response without pre-existing liver disease [1,2,3]. The primary etiologies include viral infections and drug-induced hepatoxicity [4]. ALF usually leads to coagulation dysfunction, hepatic encephalopathy, multiorgan failure, and even death [5,6,7]. Currently, the clinical treatments, including hepatoprotective drugs, microecological modulation therapy, and hormone therapy, have shown poor prognosis, frequently necessitating emergency liver transplantation [8,9,10]. Therefore, new investigation approaches are urgently needed to alleviate the disease progression and reduce the stress of liver transplantation.

The lipopolysaccharide (LPS)/D-GalNactosamine (D-GalN) has been widely utilized to investigate the pathogenesis of ALF and novel therapeutic drugs that are used to treat it [11,12,13]. LPS induced the overgeneration of inflammatory cytokines including TNF-α and IL-6 in D-GalN-sensitized mice [14,15]. The inflammatory response is pivotal in the biological pathogenesis of ALF, culminating in hepatocyte death and impaired liver function [16,17]. Thus, the modulation of the immune response and suppression of inflammation hold promising therapeutic effects on protecting the liver from ALF.

Emerging evidence has implied that the IL-33/ST2 and IL-17A signaling pathways are key regulators of inflammatory processes and potential therapeutic targets in ALF. IL-33, belonging to the IL-1 family, is secreted by endothelial cells, epithelial cells, and fibroblasts following cellular stress, necrosis, or mechanical injury [18]. IL-33 acts on a variety of immune cells, including T cells, eosinophils, dendritic cells, and macrophages [19]. It appears to be an ‘alarm’ factor in the immune system and is involved in a variety of physiologic disease processes [20]. ST2 is a selectively sheared receptor for IL-33 that exists in a full-length transmembrane form (ST2L) and a soluble secreted form (sST2). ST2L binds to IL-33 through its extracellular segment and initiates downstream signaling, whereas sST2 lacks both transmembrane and intracellular structural domains and acts as a decoy receptor for IL-33 [21,22]. The IL-33/ST2 signaling pathway plays a crucial regulatory role in inflammation responses associated with LPS-induced macrophages and dendritic cells [23,24,25]. Emerging evidence showed that sST2 injection inhibited LPS-induced IL-6 and NF-κB pathways, regulated the immune response of macrophages, followed by a reduced production of inflammatory factors, and decreased LPS-mediated mortality [26,27,28,29]. Furthermore, the study showed that the IL-33/ST2 axis played a critical role in regulating the homeostasis of Th17 cells, with sST2 administration leading to increased serum levels of IL-17A [30,31,32]. Therefore, whether sST2 treatment can ameliorate LPS/D-GalN-induced acute liver injury still requires further investigation.

IL-17A is a signature cytokine of IL-17 cytokine which is produced by Th17 cells. It binds to IL-17 RA and IL-17 RC complexes on the cell membrane surface and mediates the production of inflammatory molecules, chemokines, and antimicrobial peptides, participating in many important physiological processes such as host defense, cellular trafficking, and immune modulation [33,34,35]. Research showed that IL-17A participated in the LPS-induced pathological immune response by recruiting neutrophils, mobilizing T cells, and inducing the production of chemokines and inflammatory cytokines. On the other hand, the neutralization of IL-17A by antibodies significantly reduced the levels of inflammatory factors and chemokines in mice, providing a potential therapeutic tool [36,37,38,39,40]. Based on the interconnected roles of the IL-33/ST2 and IL-17A pathways in regulating inflammation, we hypothesized that the co-administration of sST2 and IL-17A antibodies could regulate macrophages and reduce the production of inflammatory factors, thereby providing a better immunomodulatory effect and mitigating liver injury induced by LPS/D-GalN in mice.

In this study, a novel dual-functional fusion protein, anti-IL-17A-sST2, was constructed to simultaneously block the IL-33/ST2 and IL-17A pathways. The physicochemical properties and biological activities of anti-IL-17A-sST2 were investigated. Afterward, the protective effect of anti-IL-17A-sST2 was explored on LPS/D-GalN-induced ALF mice. For mechanical studies, the regulation of the TLR4/MyD88 pathway and NLRP3 inflammasome was evaluated. Collective results indicated that co-blocking IL-33 and IL-17A could be a potential therapeutic way to treat ALF.

## 2. Materials and Methods

### 2.1. Cell Lines

RAW 264.7 and C3H/10T1/2 cells were purchased from Cell Bank (Shanghai, China) and cultured as required. 293F cell was acquired from iCareab Biotechnology Co., Ltd. (Suzhou, China) and was cultured with OPM-293 CD05 medium (81075, Opmbiosciences, Shanghai, China) and cultured as required.

### 2.2. Animals

Male C57BL/6J mice (6–8 weeks, 20–22 g) were purchased from the Shanghai Slaccas Company (Shanghai, China) and were housed as required. The proteins were administered intraperitoneally for 24 h before LPS/D-GalN injection. LPS (L4391, Sigma-Aldrich, St. Louis, MO, USA, 30 µg/kg) and D-GalN (ST1213, Beyotime Biotechnology, Shanghai, China, 600 mg/kg), dissolved in PBS, were administered intraperitoneally after preparations.

For the investigation of the therapeutic doses, anti-IL-17A-sST2 at different doses (3, 6, 12, 18, 24 mg/kg) and control antibody (control Ab, 16 mg/kg) were intraperitoneally injected into the mice. For therapeutic effect and mechanism experiment, equimolar proteins (55 nmol/kg) including anti-IL-17A (8 mg/kg), sST2-Fc (6.73 mg/kg), a combination of anti-IL-17A and sST2-Fc, anti-IL-17A-sST2 (12 mg/kg), and control Ab (8 mg/kg) were intraperitoneally injected into the mice. For the safety evaluation of anti-IL-17A-sST2, equimolar proteins (55, 110 nmol/kg) including anti-IL-17A (8, 16 mg/kg), sST2-Fc (6.73, 13.5 mg/kg), a combination of anti-IL-17A and sST2-Fc, anti-IL-17A-sST2 (12, 24 mg/kg), and control Ab (8, 16mg/kg) were intraperitoneally injected into the mice.

Blood, livers, and spleens were harvested at 4.5 h after LPS/D-GalN injection for further analysis. The body weight, liver weight, and spleen weight of the mice were recorded, and the liver index (liver weight (g)/body weight (g) × 100%) and spleen index (spleen weight (mg)/body weight (g)) were calculated. All experiments were approved by an ethics committee.

### 2.3. Vector Construction, Protein Expression, and Purification

Anti-IL-17A: the expression vectors of anti-IL-17A were based on our previous study [39], which contained the heavy-chain and light-chain variable regions of vunakizumab. sST2-Fc: the pTT5-sST2-Fc vector was constructed by cloning the DNA sequence encoding amino acids 27–332 of mouse ST2L into the pTT5 vector which contained the human IgG_4_ Fc domain. Anti-IL-17A-sST2: For the construction of vunakizumab and mouse sST2 bifunctional fusion protein, the light chain expression vector used was the pTT5-anti-IL-17A light chain vector. For the construction of the pTT5-anti-IL-17A-sST2 heavy chain vector, the DNA sequence of sST2 along with a (Gly_4_Ser)_3_ linker was fused to the C-terminus of anti-IL-17A heavy chain by polymerase chain reaction (PCR, 10136ES, YEASEN, Shanghai, China) and cloned (C115, Vazyme Biotech Co., Ltd., Nanjing, China) into the pTT5 vector, which was digested by EcoRI and BamHI (1040, 1010, TaKaRa, Shiga, Japan). Associated primers are listed in Table 1. Then, the expression vectors were manufactured into 293F cells, and the supernatants were collected 7 days after transfection, with purifying being conducted with protein A affinity columns (GE Healthcare Life Sciences, Piscataway, NJ, USA).

### 2.4. Size Exclusion Chromatography–High-Performance Liquid Chromatography (SEC-HPLC)

The SEC-TSKgel (TOSOH) column separated the protein, using PBS as the mobile phase, with the elution time being set as 40 min and the flow rate as 1.0 mL/min. Chromatographic separation was monitored via UV detection at 260 and 280 nm. Calculations were performed using the peak area normalization method.

### 2.5. Thermal Stability Analysis

Tm and Tagg values were determined using the UNCLE. SYPRO Orange (Thermo Fisher Scientific, Uppsala, Sweden) was bound to amino acid residues, and the fluorescence intensity was measured at 473 nm. The temperature was increased from 20 to 95 °C at 0.3 °C/min. The raw data obtained were fitted to a sigmoidal shape via Uncle Analysis.

### 2.6. Surface Plasmon Resonance (SPR)

The binding affinity of proteins to human IL-17A, mouse IL-17A (Sino Biological, Beijing, China), and mouse IL-33 (PDEM100058, Elabscience Biotechnology, Houston, TX, USA) was measured using the Biacore™ T200 system (GE Healthcare Life Sciences, Piscataway, NJ, USA). The antigens were immobilized on CM5 using amine coupling (GE Healthcare). Proteins were sequentially flowed through the chip in a 2-fold dilution series for 120 s with a dissociation time of 480 s. The equilibrium dissociation constant (KD) was obtained using Biacore™ T200 Analysis software 3.2.

### 2.7. In Vitro Bioactivity Assay of IL-17A

The bioactivity of IL-17A antibodies was determined by inhibition of IL-6 release. IL-17A stimulated C3H/10T1/2 cells to produce and release IL-6 into the cell supernatants. IL-17A mAb (1 nM), anti-IL-17A-sST2 (1 nM), and control Ab (1 nM) were diluted and IL-17A (1 μg/mL) antigen was added, with co-incubation being conducted at 37 °C for 1 h and then culture of the C3H/10T1/2 cells for 48 h. IL-6 in the supernatant was determined via ELISA (EK206, Multi Sciences, Hangzhou, China).

### 2.8. In Vitro Bioactivity Assay of sST2-Fc

ST2 inhibited the production of IL-6 production stimulated by LPS. Raw264.7 cells were cultured with sST2-Fc (0.5 mg/L), anti-IL-17A-sST2 (0.89 mg/L), or control Ab (0.59 mg/L) for 3 h and then LPS (1 mg/L) for 8 h. The concentrations of IL-6 were analyzed via ELISA (EK206, Multi Sciences, China). 

### 2.9. Serum Stability Analysis

The anti-IL-17A-sST2 or control Ab was added to the serum, which was obtained from C57BL/6J mice and filtrated using a 0.22 μm filter. The mixture was incubated at 37 °C in a CO_2_ incubator for 0.01 h, 6 h, 12 h, 24 h, and 48 h. The in vitro bioactivity of anti-IL-17A-sST2 was assessed.

### 2.10. Biochemical Analysis

Serum alanine aminotransferase (ALT) and aspartate aminotransferase (AST) activities were analyzed with detection kits (Jiancheng Bioengineering, Nanjing, China). 

### 2.11. Western Blot

Liver total proteins were quantified via BCA (Yeasen). Each well plate contained about 20 µg protein samples, and the antibodies included MyD88 (bs-1047R, Bioss, Beijing, China), caspase-1 (ab179515, Abcam, Cambridge, UK), and GAPDH (2118S, Cell Signaling Technology, Boston, FL, USA). Then, the corresponding secondary antibody was selected according to the source of the primary antibody (Thermo Fisher Scientific, Uppsala, Sweden). The target bands were visualized with the electrochemiluminescence reagent (Meilunbio, Dalian, China) and analyzed via Image J software V1.8.0. 

### 2.12. RT-qPCR

Total RNA was measured by nanodrop, reversed to cDNA (R333, Q711, Vazyme Biotech Co., Ltd., Nanjing, China), and analyzed on QuantStudio 3 (Thermo Fisher Scientific, Uppsala, Sweden). Specific primers are listed in Table 2. 

### 2.13. Hematoxylin and Eosin (H&E) Staining 

Fresh tissue was fixed, embedded, sectioned, stained, and photographed under SlideView VS200 (Olympus, Tokyo, Japan). 

### 2.14. Immunohistochemistry (IHC) Assay

The antibodies included TLR4 (1:100, GB11519, Servicebio, Wuhan, China) and NLRP3 (1:100, CY5651, Abways Technology, Shanghai, China). The images were taken by Sideview VS200 (Olympus, Japan) and were quantified with Image J.

### 2.15. Statistical Analysis

All data were visualized as mean ± SD using Graph Pad Prism 9.0 software. Student’s *t*-test or one-way ANOVA was used to compare the differences. * *p* < 0.05, ** *p* < 0.01, ns, not significant.

## 3. Results

### 3.1. Generation and Characterization of the Fusion Protein of IL-17A Antibody and sST2

**Figure 1:**
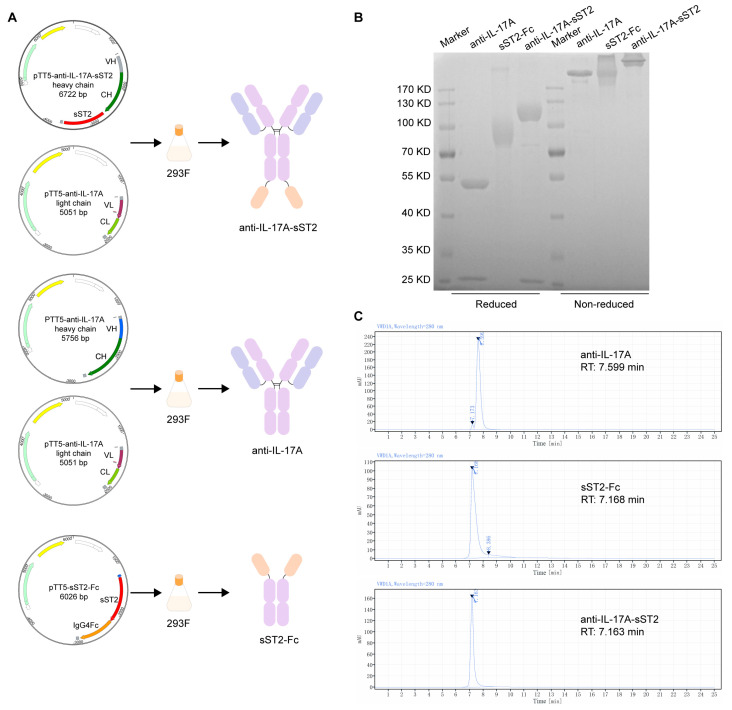
Expression, purification, and characterization of anti-IL-17A-sST2. (**A**) Schematic representation of generation and expression of proteins. (**B**) Reduced and non-reduced SDS-PAGE analysis of proteins after affinity purification. (**C**) SEC-HPLC analysis of proteins. (RT: retention time).

The gene sequence encoding sST2 was linked to the C-terminus of IL-17A mAb via a flexible linker (Gly_4_Ser)_3_. The resulting plasmid, together with the pTT5-anti-IL-17A light chain vector, which contained the gene encoding the light chain of anti-IL-17A, was used in the production of the anti-IL-17A-sST2 fusion protein (Figure 1A). SDS-PAGE and SEC-HPLC were used to assess the purities of anti-IL-17A, sST2-Fc, and anti-IL-17A-sST2. The SDS-PAGE image showed that anti-IL-17A-sST2 had bands at the expected molecular weight, which had a larger heavy band than the anti-IL-17A (Figure 1B). SEC-HPLC analysis showed purity levels of 97.28%, 93.64%, and 100% for IL-17A mAb, sST2-Fc, and anti-IL-17A-sST2, respectively, suggesting that the proteins had high purity (Figure 1C).

Melting temperature (Tm) and aggregation temperature (Tagg) are fundamental parameters for measuring protein stability. Tm is the temperature at which an antibody molecule transitions from its native folded state to a fully unfolded state. Tm1 and Tm2 typically represent different transitions or domains within the Fab and Fc structures. Tm and Tagg were accessed via intrinsic protein fluorescence measurements (Table 3). The Tm1 values of anti-IL-17A-sST2 were 68.41 °C, indicating good thermal stability. According to the signal change of SLS 266 or SLS 473, the Tagg 266 and Tagg 473 of anti-IL-17A-sST2 were 67.67 °C and 66.65 °C, respectively. The Tm1 and Tagg values of anti-IL-17A-sST2 were close, suggesting that the aggregation and denaturation of anti-IL-17A-sST2 might occur together. 

### 3.2. The Characterization and Bioactivity of the Fusion Protein

**Figure 2:**
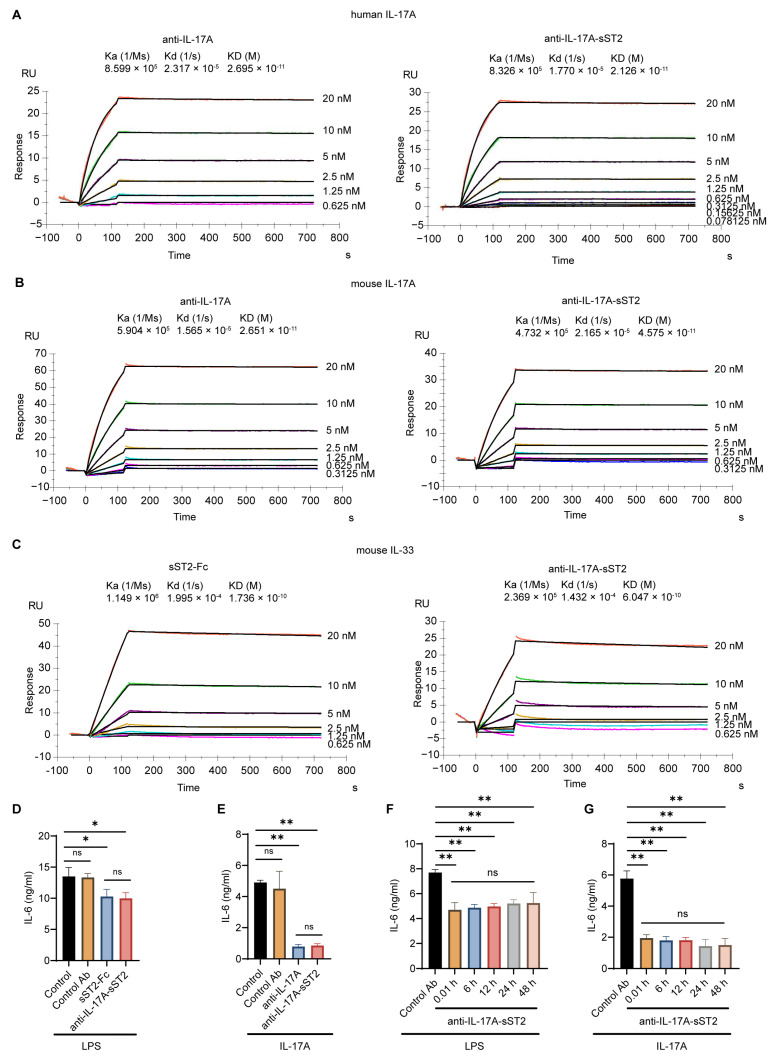
Affinity and bioactivity of anti-IL-17A-sST2. Affinity analysis of IL-17A mAb and anti-IL-17A-sST2 for human IL-17A antigen (**A**) and mouse IL-17A antigen (**B**) measured by SPR. (**C**) Affinity analysis of sST2-Fc and anti-IL-17A-sST2 for mouse IL-33 antigen measured by SPR. (**D**) The in vitro inhibition by anti-IL-17A-sST2 and sST2-Fc on LPS-induced release of IL-6 from Raw 264.7 (n = 3). (**E**) The in vitro inhibition by anti-IL-17A-sST2 and IL-17A mAb on IL-17A caused IL-6 secretion from C3H10T1/2 (n = 3). (**F**) The inhibition by anti-IL-17A-sST2 fusion protein on LPS-induced release of IL-6 from Raw 264.7, which was incubated in serum for 0.01 h, 6 h, 12 h, 24 h, and 48 h (n = 4). (**G**) The inhibition by anti-IL-17A-sST2 fusion protein on IL-17A caused IL-6 secretion from C3H10T1/2, which was incubated in serum for 0.01 h, 6 h, 12 h, 24 h, and 48 h (n = 4). The data in (**D**–**G**) represent means ± SDs. * *p* < 0.05, ** *p* < 0.01, ns, not significant. Significant differences were determined by one-way ANOVA.

The binding affinity was evaluated. The SPR results exhibited that the affinity of the anti-IL-17A-sST2 fusion protein for human IL-17A and mouse IL-17A was similar to anti-IL-17A (Figure 2A,B). Anti-IL-17A-sST2 had excellent affinity to human IL-17A at 0.02126 nM and mouse IL-17A at 0.04575 nM, whereas anti-IL-17A displayed affinity to human IL-17A at 0.02695 nM and mouse IL-17A at 0.02651 nM. These data suggest that the presence of sST2 did not affect the binding ability of fusion protein for binding to IL-17A. Meanwhile, the affinity of anti-IL-17A-sST2 and sST2-Fc to mouse IL-33 antigen was also comparable (Figure 2C) with the affinity to mouse IL-33 at 0.6047 nM and 0.1736 nM for anti-IL-17A-sST2 and sST2-Fc, respectively.

Then, the bioactivity of anti-IL-17A-sST2 was evaluated in vitro. The inhibitory effect of ST2 was assessed by analyzing the release of LPS-induced IL-6 on Raw264.7 cells [41]. ELISA results demonstrated that anti-IL-17A-sST2 inhibited IL-6 secretion similarly to sST2-Fc (Figure 2D). The inhibition function of the fusion protein against IL-17A was examined by analyzing the IL-6 secretion from C3H10T1/2 cells caused by IL-17A [42,43,44]. As shown in Figure 2E, anti-IL-17A-sST2 effectively suppressed the IL-6 secretion in C3H10T1/2 cells, which was comparable to anti-IL-17A. These experiments indicated that anti-IL-17A-sST2 possessed the biological functions of both anti-IL-17A and sST2-Fc. Furthermore, after incubation with serum for 6 h, 12 h, 24 h, and 48 h, the bioactivity of anti-IL-17A-sST2 showed no significant change, indicating good serum stability of anti-IL-17A-sST2 (Figure 2F–G). 

### 3.3. Anti-IL-17A-sST2 Ameliorated the Liver Injury Induced by LPS/D-GalN 

**Figure 3:**
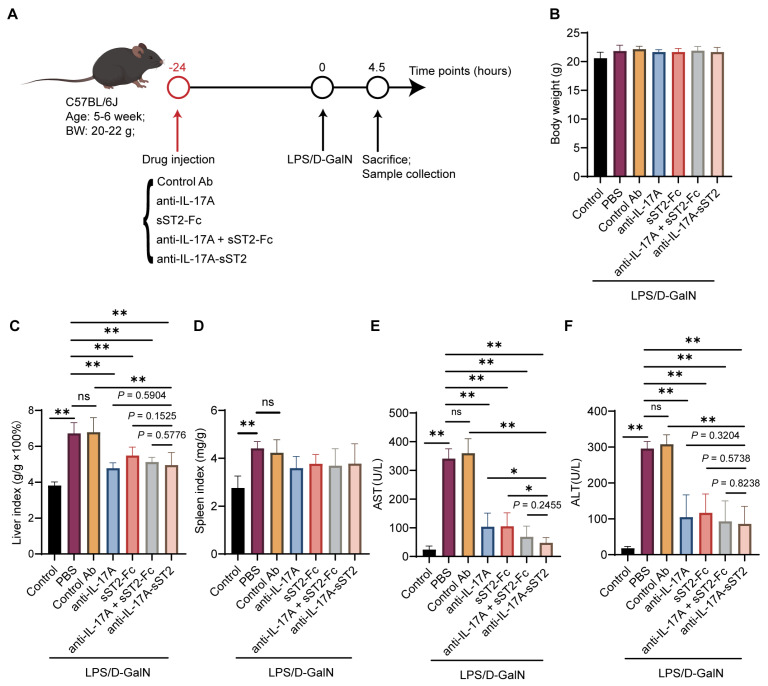
The therapeutic effect of anti-IL-17A-sST2 against LPS/D-GalN-induced liver injury. (**A**) Schematic illustration for experimental design of the protective effect of anti-IL-17A-sST2 in LPS/D-GalN-induced ALF mice. Mice were injected by intraperitoneal injection with proteins 24 h in advance; then, they were injected with LPS/D-GalN and sacrificed after 4.5 h. (**B**) Quantitative analysis of body weight of mice with indicated injection (n = 6). (**C**) Liver index × 100% (liver weight/body weight, g/g × 100) (n = 6). (**D**) Spleen index (spleen weight/body weight, mg/g) (n = 6). (**E**,**F**) Levels of AST and ALT. The data in (**B**–**F**) represent means ± SDs. * *p* < 0.05, ** *p* < 0.01, ns, not significant; *p*-values were determined by Student’s *t*-test.

The range of doses at which anti-IL-17A-sST2 produced therapeutic effects was investigated. The administration of 12, 18, and 24 mg/kg anti-IL-17A-sST2 exhibited a significant palliative effect on AST and ALT levels in LPS/D-GalN-induced ALF mice (Supplementary Figure S1A,B). A dose of 12 mg/kg was chosen for subsequent experiments. The groups of mice were injected with IL-17A mAb, sST2-Fc, the combination of both, or anti-IL-17A-sST2 24 h prior. Subsequently, the mice were injected with LPS/D-GalN for 4.5 h and then euthanized (Figure 3A). Serums, livers, and other organs were harvested for further analysis. No significant changes in the body weight of mice were observed during this process (Figure 3B). 

The livers exhibited blackening with hemorrhaging and congestion, while spleens were blackened and enlarged after LPS/D-GalN injection (Supplementary Figure S2). The above treatments improved the severity of apparent lesions, as well as liver and spleen congestion and enlargement in mice, and the livers of mice treated with fusion protein exhibited improvements. The LPS/D-GalN-induced mice had an increased liver and spleen index (Figure 3C,D), and a lower liver index could be detected in mice after anti-IL-17A, sST2-Fc, a combination of both, and anti-IL-17A-sST2 administration. Meanwhile, the LPS/D-GalN injection significantly increased serum AST and ALT levels, suggesting that it impairs hepatic function (Figure 3E,F). AST and ALT levels were significantly reduced after anti-IL-17A-sST2 treatment compared to PBS or control Ab treatment. Notably, the AST level after anti-IL-17A-sST2 treatment was half of the AST level after treatment with anti-IL-17A alone or sST2-Fc alone, and it slightly outperformed their combination treatment, indicating that it exhibits a better function of modulating both IL-17A and IL-33/ST2 pathways in liver protection. These results demonstrated that anti-IL-17A-sST2 could ameliorate liver injury, as well as improve liver function.

### 3.4. Anti-IL-17A-sST2 Improved the Histopathological Changes and Reduced Inflammatory Factors

**Figure 4:**
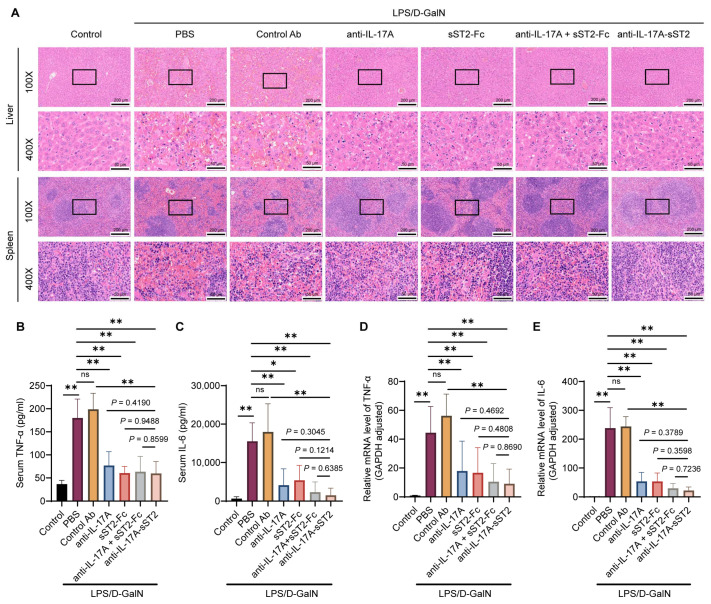
Attenuation of liver injury and inflammation following treatment with anti-IL-17A-sST2. (**A**) Representative H&E staining images of liver and spleen tissues with indicated treatments. (Magnification: 100×, 40 ×). Scale bar = 200 μm (100 ×) and 50 μm (400 ×). (**B**,**C**) TNF-α and IL-6 levels in serum samples (n = 4). (**D**,**E**) Relative mRNA expression of liver TNF-α and IL-6 (n = 4). The data in (**B**–**E**) represent means ± SDs. * *p* < 0.05, ** *p* < 0.01, ns, not significant; *p*-values were determined by Student’s *t*-test.

The hepatic and splenic tissues from various groups underwent H&E staining. Hepatic images from control Ab groups showed obvious pathological alterations, which were primarily characterized as hepatocyte necrosis and interstitial hemorrhage (Figure 4A). Anti-IL-17A-sST2 displayed reduced hemorrhaging and structural disorders compared to the control Ab group, as well as IL-17A mAb or sST2-Fc alone, which were consistent with the splenic tissues. These results suggested that the anti-IL-17A-sST2 injection provided enhanced protection under the LPS/D-GalN challenge.

LPS/D-GalN injection significantly elevated serum and liver tissue TNF-α and IL-6 levels. Anti-IL-17A-sST2 administration significantly reduced the TNF-α and IL-6 levels compared to the PBS or control Ab group (Figure 4B,C). The TNF-α level after anti-IL-17A-sST2 treatment was comparable to the levels following treatment with IL-17A mAb, sST2-Fc, or the combination, while the level of IL-6 after anti-IL-17A-sST2 treatment was lower than IL-17A mAb, sST2-Fc, or the combination injection. Similarly, *TNF-α* and *IL-6* mRNA expression were significantly suppressed after anti-IL-17A-sST2 injection compared to the PBS or control Ab group, which was lower than the treatment with IL-17A mAb, sST2-Fc, or the combination (Figure 4D,E). Thus, these results exhibited that anti-IL-17A-sST2 treatment could significantly inhibit inflammatory mediator secretion and attenuate inflammatory responses.

### 3.5. Anti-IL-17A-sST2 Decreased TLR4 Expression in the Liver

**Figure 5:**
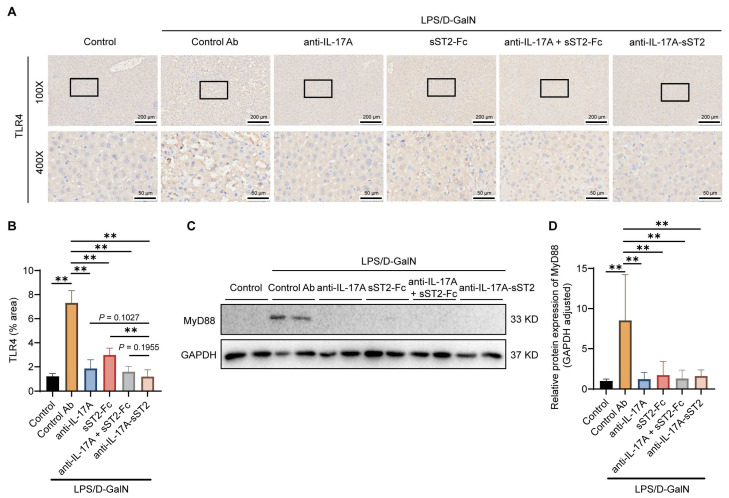
Downregulation of the TLR4/MyD88 pathway in liver tissue following anti-IL-17A-sST2 treatment. (**A**) Representative images of immunohistochemistry analysis of TLR4 in liver tissues. (Magnification: 100×, 400×). Scale bar = 200 μm (100×) and 50 μm (400×). (**B**) Analysis of TLR4 in liver sections (n = 6). (**C**,**D**) Western blot analysis of MyD88 in mice livers with indicated treatments, using GAPDH as the control (n = 4). The data in (**B**,**D**) represent means ± SDs. ** *p* < 0.01; *p*-values were determined by Student’s *t*-test.

To elucidate the relevant mechanistic pathways, the signaling pathways and important molecules associated with LPS-mediated inflammation were investigated. Several studies have shown that LPS can activate macrophages in liver tissue, leading to the secretion of inflammatory cytokines and inducing hepatocyte necrosis or apoptosis. Activation of Toll-like receptor 4 (TLR4) occurs upon exposure to LPS, leading to the activation of its downstream signaling pathway and subsequent induction of a pro-inflammatory response [45]. Because the treatment with PBS and control Ab exhibited no significant differences in liver injury, AST and ALT levels, or inflammatory factor levels, the control Ab treatment was used as a control in the subsequent experiments. The IHC results of TLR4 in mice livers showed that anti-IL-17A-sST2 downregulated the expression of TLR4 in the liver compared to the control Ab group (Figure 5A,B). The level of TLR4 under anti-IL-17A-sST2 injection was significantly downregulated compared to the sST2-Fc injection and was marginally reduced compared to the anti-IL-17A or the combination injection. Meanwhile, the level of MyD88, the downstream signaling molecule of TLR4, was significantly decreased after anti-IL-17A-sST2 injection (Figure 5C,D). The results indicated that anti-IL-17A-sST2 might mitigate the hepatic inflammatory response by suppressing the TLR4/MyD88 signal.

### 3.6. Anti-IL-17A-sST2 Suppressed NLRP3 Inflammasome Activation

**Figure 6:**
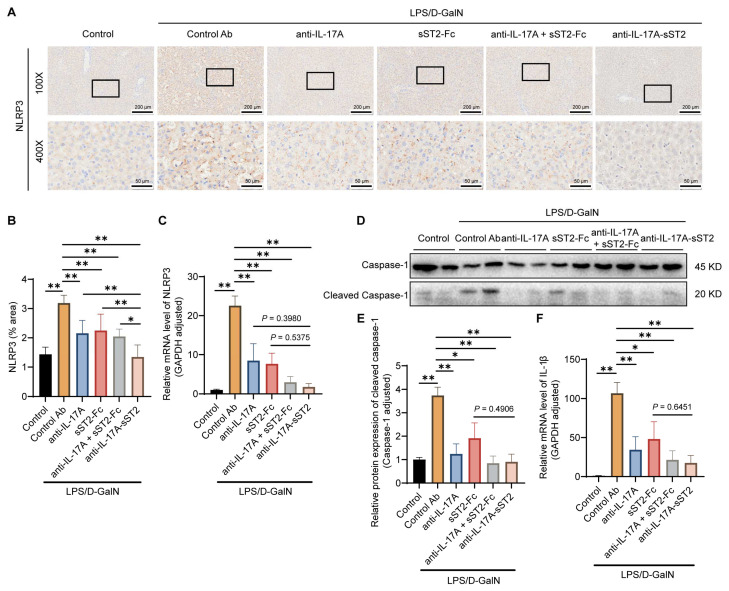
Inhibition of NLRP3 inflammasome activation following anti-IL-17A-sST2 injection. (**A**) Representative images of immunohistochemistry analysis of TLR4 in liver tissues. (Magnification: 100×, 400×). Scale bar = 200 μm (100×) and 50 μm (400×). (**B**) Analysis of TLR4 in liver sections (n = 6). (**C**) The NLRP3 mRNA expression in the mice liver with indicated treatment (n = 4). (**D**,**E**) Western blot analysis of Caspase-1/Cleaved Caspase-1 in mice livers with indicated treatments (n = 4). (**F**) The mRNA expression of IL-1β in mice livers with indicated treatment (n = 4). The data in (**B**,**C**,**E**,**F**) represent means ± SDs. * *p* < 0.05, ** *p* < 0.01; *p*-values were determined by Student’s *t*-test.

The TLR4 signal promotes NLRP3 inflammatory activation, an important inflammatory mediator, initiating the transcription and expression of IL-1β, which promotes hepatocyte pyroptosis [46]. As shown in Figure 6A,B, the injection of anti-IL-17A-sST2 significantly reduced the number of cells with positive staining of NLRP3 compared to the control Ab group, as well as those treated with anti-IL-17A, sST2-Fc, or the combination. The mRNA expression of NLRP3 was also downregulated after anti-IL-17A-sST2 treatment, which was significantly reduced compared to the control Ab group and was lower than the anti-IL-17A, sST2-Fc, or the combination group (Figure 6C). Meanwhile, the activation of cleaved caspase-1 and the transcription of *IL-1β* were significantly downregulated after anti-IL-17A-sST2 treatment compared to the control Ab group, which was also lower than the other treatment (Figure 6D–F). These results indicated that fusion protein reduced the activation of the NLRP3 inflammasome and the production of inflammatory factors.

### 3.7. Anti-IL-17A-sST2 Appeared No Significant Toxicity on the Main Organs

To evaluate the safety of anti-IL-17A-sST2, H&E staining was performed on the major organs of mice treated with anti-IL-17A, sST2-Fc, the combination, or anti-IL-17A-sST2 of two different doses (Supplementary Figure S2A,B), in which no significant pathological damage was found. These results suggested that fusion proteins exhibit low toxicity.

## 4. Discussion

ALF is a clinicopathological syndrome, which is characterized by a systemic inflammatory response and massive hepatocyte death [1]. Traditional therapy falls short, highlighting the urgent need for new therapeutic tools. In LPS/D-GalN-induced ALF mice, LPS stimulates macrophages via pattern recognition receptors, which activate and secrete inflammatory agents, further inducing hepatocyte apoptosis and chronic inflammation. IL-17A and IL-33/ST2 have critical regulatory roles in the immune response and inflammatory diseases. sST2 treatment inhibits LPS-induced macrophage and dendritic cell activation, whereas IL-17A recruits neutrophils and mobilizes T cells, promoting the production of chemokines and inflammatory cytokines. Herein, a novel bifunctional fusion protein, anti-IL-17A-sST2, was constructed to simultaneously target these pathways. The results showed that anti-IL-17A-sST2 administration decreased hepatocyte death, suppressed an excessive inflammatory reaction, and reduced the secretion of pro-inflammatory mediators, indicating that anti-IL-17A-sST2 might be the potential therapeutic approach for ALF.

The reciprocal regulatory relationship between the IL-17A and IL-33/ST2 signals is pivotal in regulating the immune response [47]. IL-33 is present in the nucleus as a precursor and possesses transcriptional regulatory properties—which regulate pro-inflammatory signals—and chemokines—which modulate the immune response [48,49,50]. Upon a tissue injury, IL-33 is proteolytically cleaved into a highly active form and released extracellularly, where it binds to the ST2L-IL1RAP complex, thereby activating downstream pathways and stimulating the release of inflammatory factors [23,29,51]. Therefore, sST2 can inhibit the IL-33-induced immune response by competitively binding to the molecule [52]. However, IL-33 is not only involved in the processes of the Th2-associated immune response but also in the regulation of the equilibrium between Th17 and Treg. In allergic airway inflammation, IL-33 synergistically enhances neutrophil inflammation with IL-17A through the CXCR2 pathway [32,53]. IL-17A plays a proinflammatory role in several immune disorders, and IL-17A-blocking antibodies can effectively alleviate several diseases, including arthritis [54]. The aforementioned evidence show that IL-17A stimulated the generation of chemokines and attracted neutrophils together with other pro-inflammatory factors. Research exhibited that IL-17A participated in the process of LPS-induced inflammatory response and that the IL-17A antibody reduced the mortality rate of neonatal sepsis [36,55,56]. Pre-treatment with the IL-17A antibody could attenuate liver injury and reduce neutrophil infiltration induced by triptolide/LPS, which is associated with a Th17/Treg imbalance [57]. Given this intricate relationship between IL-17A and IL-33/ST2, we validated that the simultaneous modulation of the IL-33/ST2 and IL-17A pathways might be a promising therapeutic option. 

The fusion protein, anti-IL-17A-sST2, was constructed to simultaneously modulate the IL-33/ST2 and IL-17A pathways. The injection of anti-IL-17A-sST2 significantly reduced serum AST levels and downregulated the expression of TLR4 and NLRP3, compared to the injection with anti-IL-17A or sST2-Fc alone. These results indicate that targeting both IL-33/ST2 and IL-17A pathways provides a better protective efficacy in ameliorating liver injury and suppressing the inflammatory response in LPS/D-GalN-induced ALF mice. Notably, anti-IL-17A-sST2 was more effective in suppressing NLRP3 inflammasome activation compared to the combination of anti-IL-17A and sST2-Fc, as evidenced by the decrease in NLRP3-positive cells. Moreover, anti-IL-17A-sST2 treatment could further reduce AST levels compared to combination treatment, indicating that it provide better protection for liver function in LPS/D-GalN-induced mice. In addition to its therapeutic benefits, anti-IL-17A-sST2 had the potential to extend the half-life of sST2 and reduce production costs. Therefore, the administration of anti-IL-17A-sST2 might be a more favorable therapeutic approach for concurrently modulating the IL-33/ST2 and IL-17A pathways. Meanwhile, this study has some limitations. In our results, only LPS/D-GalN-induced ALF mice were used to investigate the therapeutic effects of anti-IL-17A-sST2, in which their injuries progressed rapidly, and the macrophages played a critical role in the inflammation response. The swift development of the disease in this model may have restricted some of the drug’s therapeutic effects, resulting in less pronounced differences in some of the metrics, such as the level of TNF-α, in anti-IL-17A-sST2 compared to the individual components and their combination in addition to the relevant results above. Further research could concentrate on structural improvements and the exploration of alternative inflammatory disease models, thereby deepening our understanding of the therapeutic potential of anti-IL-17A-sST2.

Anti-IL-17A-sST2 fusion protein was constructed by fusing the sST2 to the C-terminus of IL-17A mAb via a flexible linker (Gly_4_Ser)_3_, which resulted in a homodimer structure. The homodimer is one of the most commonly used formats for designing antibody–cytokine fusion. Our prior research demonstrated that integrating sST2 at the C-terminus of the antibody resulted in better performance than integration at the N-terminus. Interestingly, studies have suggested that although the homodimer often exhibited high affinity and bioactivity, the heterodimer may have additional advantages [58]. Therefore, in future phases, the construction of fusion proteins can be optimized by experimenting with different structural forms and comparing their differences to determine the optimal combination. 

LPS is a major ligand for TLR4, a pattern recognition receptor. Once attached to LPS, TLR4 recruits MyD88 and other related bridging molecules to form an intracellular signaling complex that activates downstream signaling [45]. Research has demonstrated that after an LPS injection, TLR4 is increased and involved in inflammatory cytokine generation, the inflammation response, and tissue damage. Inhibitors targeting TLR4 can reduce cell death and improve organ function by blocking the LPS/TLR4 pathway [59,60]. The impact of anti-IL-17A-sST2 on hepatic TLR4 and MyD88 levels was evaluated. The results revealed that anti-IL-17A-sST2 significantly reduced hepatic TLR4 levels, suppressed the TLR4/MyD88 pathway, and alleviated the LPS-induced release of inflammatory factors.

The activation of NLRP3 inflammasome plays a vital role in exacerbating inflammation. In hepatocytes, NLRP3 inflammasome can recruit pro-caspase-1 and caspase-1 activation. Caspase-1 cleaves inactive proIL-1β into mature IL-1β [46]. The NLRP3 inflammasome is implicated in the regulation of the inflammatory reaction and is associated with hepatocyte death in ALF mice [61,62,63]. The results showed that anti-IL-17A-sST2 treatment inhibited the NLRP3/caspase-1 pathway, as evidenced by the downregulation of NLRP3 levels, and cleaved caspase-1 expression and IL-1β transcript levels in mice livers, suggesting that it caused a reduction in cell death and inflammatory mediator release.

In conclusion, this study successfully constructed an anti-IL-17A-sST2 dual-functional fusion protein by fusing sST2 to the C-terminus of the IL-17A antibody, demonstrating its excellent purity. Biacore analysis revealed that the affinity of anti-IL-17A-sST2 for the human or mouse IL-17A antigen was comparable to anti-IL-17A and that the affinity for the mouse IL-33 antigen was comparable to sST2-Fc. The protein exhibited biological functions corresponding to both anti-IL-17A and sST2, suggesting that the fusion strategy was feasible. The in vivo results revealed a synergistic anti-inflammatory effect of anti-IL-17A-sST2, which significantly improved the LPS/D-GalN-induced ALF in mice. The liver and spleen morphologies were notably enhanced, alongside a substantial decrease in the liver index and significant reductions in the circulating levels of ALT and AST. Mechanistic studies showed that anti-IL-17A-sST2 downregulated the TLR4/MyD88 pathway and NLRP3 inflammasome activation. These findings provide new potential therapeutic agents for the treatment of ALF and offer insights into novel treatment strategies for this condition.

## Figures and Tables

**Table 1:** Primer pairs used for vector construction.

Primer	Sequence (5′→3′)
sST2-F	AGGAAGCGGAGGAGGAGGAAGCGGAGGAGGAGGATCTAGCAAGAGCTCTTGGGGCC
sST2-R	GAGGTCGAGGTCGGGGGATCCCTACTACCGGTGGTCGATAGGCTG
IL-17A-F	AAACGGATCTCTAGCGAATTCG
IL-17A-R	TTCCTCCTCCTCCGCTTCCTCCTCCTCCTCCCAGAGACAGAGACAGGCTCTTCTGG

**Table 2:** Primer pairs used for PT-qPCR.

Primer	Sequence Forward	Sequence Reverse
GAPDH	GTCCTCAGTGTAGCCCAAGATG	CAATGTGTCCGTCGTGGATCT
TNF-α	GCCACCACGCTCTTCTGTCT	GGTCTGGGCCATAGAACTGATG
IL-6	CTCATTCTGCTCTGGAGCCC	TGCCATTGCACAACTCTTTTCT
NLRP3	CCCTTGGAGACACAGGACTC	GAGGCTGCAGTTGTCTAATTCC
IL-1β	ACCTGTGTCTTTCCCGTGG	TCATCTCGGAGCCTGTAGTG

**Table 3:** Thermal stability analysis of anti-IL-17A-sST2.

Sample	Tm1 (°C)	Tonset (°C)	Tm2 (°C)	Tagg 266 (°C)	Tagg 473 (°C)
anti-IL-17A-sST2	68.41	57.95	78.24	67.67	66.65
anti-IL-17A	72.01	61.83	/	65.76	69.49
sST2-Fc	81.10	68.79	/	73.31	70.89

## Data Availability

The authors confirm that all data underlying the findings are fully available without restrictions.

## Supplementary Materials

The following supporting information can be downloaded at: https://www.mdpi.com/article/10.3390/biomedicines12051118/s1, Supplementary Figure S1. Evaluation of the therapeutic dose of anti-IL-17A-sST2. Supplementary Figure S2. Representative images of livers and spleens of mice with indicated injections. Supplementary Figure S3. Evaluation of medication safety of anti-IL-17A-sST2 in various tissues.
