# Supplementary material for: A Novel Bifunctional Fusion Protein (Anti-IL-17A-sST2) Protects against Acute Liver Failure, Modulating the TLR4/MyD88 Pathway and NLRP3 Inflammasome Activation
Source: Biomedicines. 2024 May 17;12(5):1118. doi: 10.3390/biomedicines12051118 (PMC11117730; doi:10.3390/biomedicines12051118)
Supplement: Supplementary file 1 [file biomedicines-12-01118-s001.zip › biomedicines-2955613-supplementary.pdf]

biomedicines

MDPI

Supplementary information

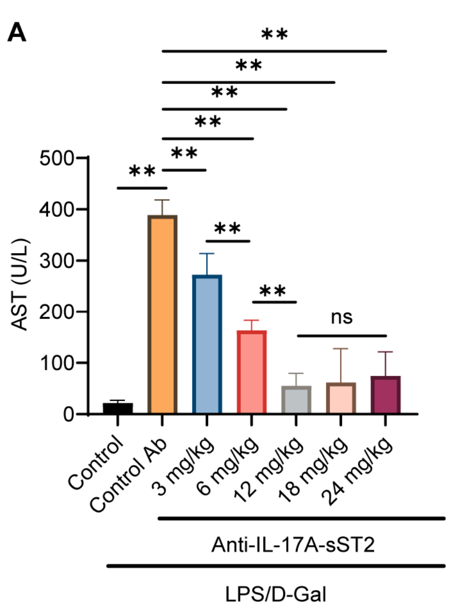

Supplementary Figure S1. Evaluation of the therapeutic dose of anti-IL-17A-sST2. (A) The levels of AST after indicated treatments (n = 4). (B) The levels of ALT after indicated treatments (n = 4). The data in (A) and (B) represent means ± SD. ***P* < 0.01, ns, not significant, were determined by one-way ANOVA.

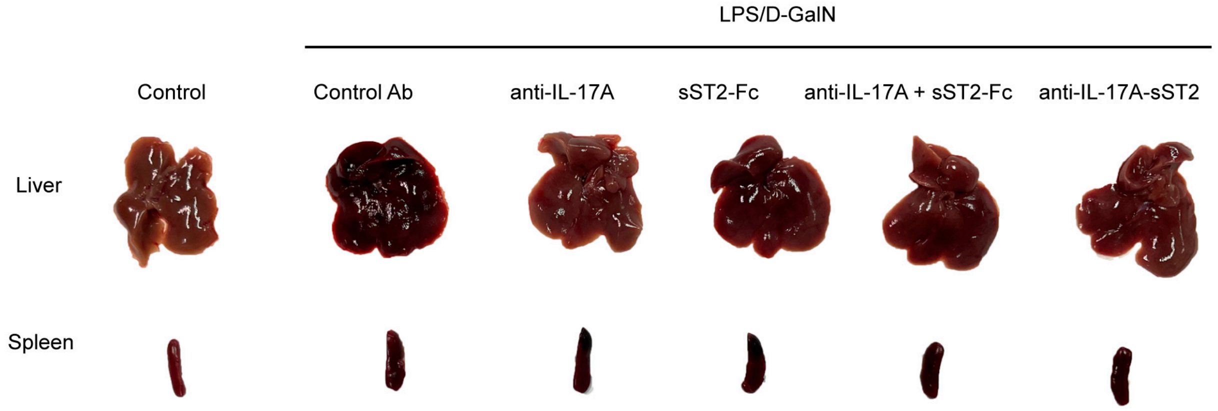

Supplementary Figure S2. Representative images of livers and spleens of mice with indicated injection.

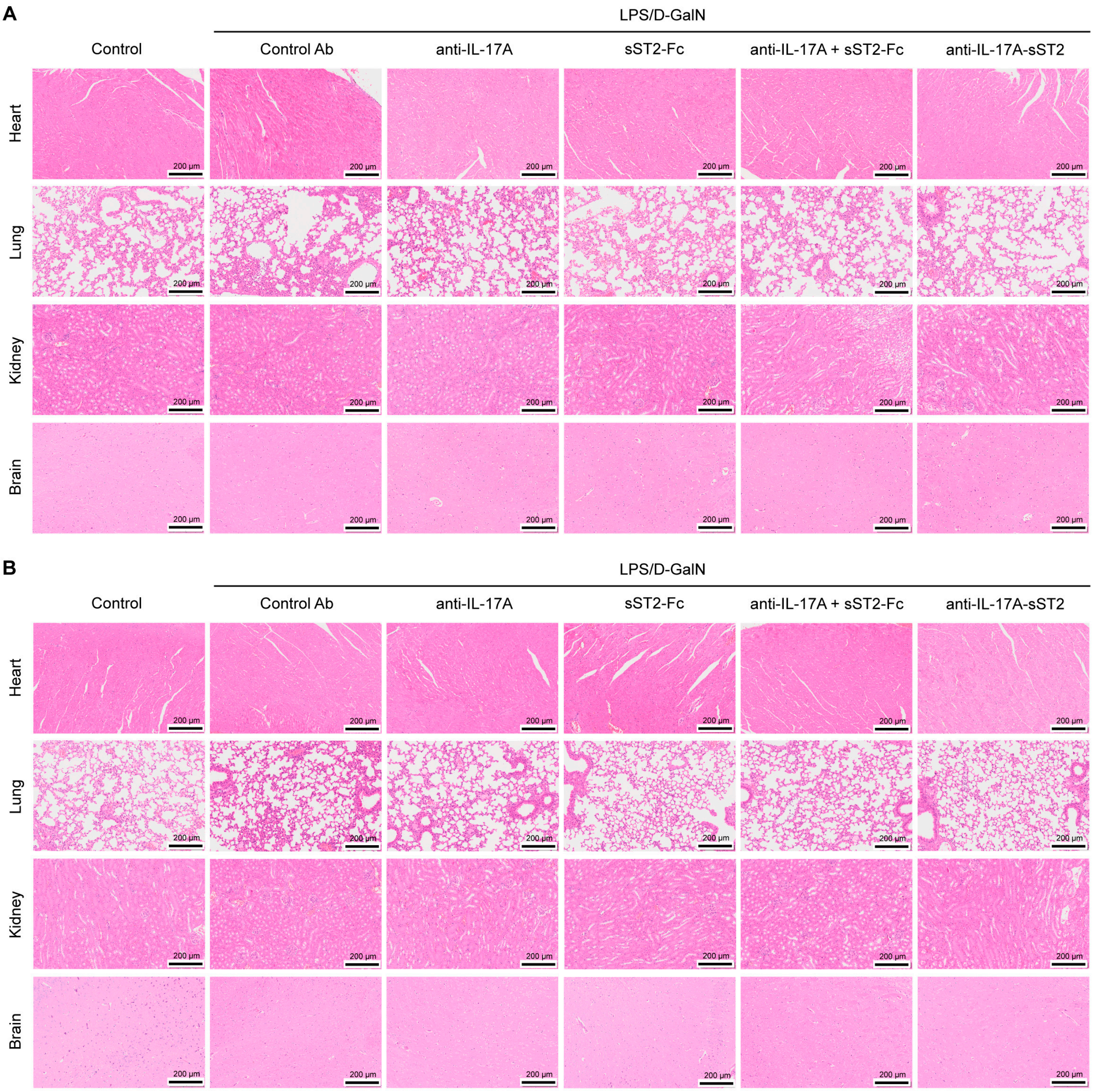

Supplementary Figure S3. Evaluation of medication safety of anti-IL-17A-sST2 in various tissues. (A) Representative H&E staining images of heart, lung, kidney, and brain tissues with indicated treatments, which anti-IL-17A (8 mg/kg) or equimolar proteins including sST2-Fc (6.73 mg/kg), anti-IL-17A-sST2 (12 mg/kg), and control Ab (8 mg/kg) were intraperitoneally injected into the mice (magnification: 100 ×, 400 ×). Scale bar = 200 μm (100 ×) and 50 μm (400 ×). (B) Representative H&E staining images of heart, lung, kidney, and brain tissues with indicated treatments, which anti-IL-17A (16 mg/kg) or equimolar proteins including sST2-Fc (13.5 mg/kg), anti-IL-17A-sST2 (24 mg/kg), and control Ab (16 mg/kg) were intraperitoneally injected into the mice (magnification: 100 ×, 400 ×). Scale bar = 200 μm (100 ×) and 50 μm (400 ×).
